# Disparities in the Diagnosis of Hypertrophic Obstructive Cardiomyopathy: A Narrative Review of Current Literature

**DOI:** 10.1155/2018/3750879

**Published:** 2018-10-02

**Authors:** Joseph Burns, Philippe Jean-Pierre

**Affiliations:** ^1^Florida International University, Herbert Wertheim College of Medicine, Miami, FL 33199, USA; ^2^Florida International University, Department of Biological Sciences, Miami, FL 33199, USA

## Abstract

Hypertrophic obstructive cardiomyopathy (HOCM) is a disorder of abnormal thickening of the myocardium that affects 0.2% of the population. HOCM is a frequently implicated cause of sudden cardiac death (SCD) in young athletes. In this manner, this condition has the capacity for tremendous emotional, social, financial, and medical burdens for families and communities across the country. Multiple factors including genetics and hormonal elements are believed to play a role in the development of this cardiomyopathy. HOCM is an autosomal dominant trait with variable expressivity. It is associated with several genetic changes in the myosin heavy chain genes. Current treatment includes optimization of cardiac risk factors and medical management with beta-adrenergic blockade with definitive treatment of surgical intervention to reduce cardiac workload. The demographic most likely to be diagnosed with HOCM based upon clinical registry data is Caucasian boys and men. However, a growing body of data supports increased prevalence in African American populations and percentages equal to, if not higher than, Caucasian males in Hispanic populations. Similarly, males of African American ethnicity that participate in basketball are the most likely demographic to be affected by HOCM based on the data available from the National Collegiate Athletic Association (NCAA). Further, though rates of diagnosis may be up to 1.5 times higher in males than in females, an increasing number of studies demonstrate an increased prevalence of HOCM in females, often presenting with worse symptoms and an increased incidence of disease progression. Similarly, data suggest that age of diagnosis is associated with various prognostic factors including annual mortality. In addition, consideration of the social determinants of health undoubtedly impacts the rate of diagnosis, access to care, and HOCM-related complications in underserved populations. Effective screening including auscultation and electrocardiography (EKG) with confirmatory echocardiography in these communities supports equitable surveillance and management of HOCM.

## 1. Introduction

Hypertrophic obstructive cardiomyopathy (HOCM) is an inherited disorder of the myocardium present in roughly 0.2% of the population [1]. In this condition, the myocardium of the ventricular walls or septum is thickened to a pathological degree. Frequently, HOCM is defined as thickness of either ventricle larger than 15 mm [2]. This anatomy can be visualized by ultrasound. In this state, the musculature of the heart impedes flow of blood through the ventricular outflow tract, resulting in increased systolic pressures and cardiac work. Persistently stressed hearts, as is the case in HOCM, are subject to increased frequency of fatal arrhythmias, cardiac arrest, and sudden death [3].

HOCM is frequently associated with sudden cardiac death (SCD) in young athletes [4]. Based on data available from the National Collegiate Athletic Association, the overall risk of SCD in athletes is one in 53,704 athlete-years [5]. However, further evaluation reveals that among this population, male gender, African American ethnicity, and basketball participation place athletes at a higher risk [5]. On the contrary, in this dataset, female gender and white or Hispanic ethnicity demonstrate lower risk than that of the overall population. However, because of its variable expression and clinical course, individuals diagnosed with HOCM may have a largely typical life with normal lifespan [6]. It is estimated that the annual mortality of all individuals affected by HOCM may be as high as 6% [7].

As a cause of SCD, HOCM is a source of tremendous emotional, social, financial, and medical burden. The loss of an outwardly healthy young person without warning has the potential for unspeakable upheaval for families and communities across the United States. Though the body of data regarding HOCM is growing, our understanding of this disease process and its implications remains incomplete. Thus, it is critical to identify populations in which the opportunity for diagnosis or screening may be missed. It is the objective of this literature review to discuss the groups most likely affected by disparities in identification of this disorder in order to prepare current and future providers to serve all of those affected by HOCM.

## 2. Hypertrophic Obstructive Cardiomyopathy

### 2.1. Genetics of HOCM

HOCM is an autosomal dominant trait with variable expressivity [8]. To date, this disorder remains the most common hereditary cardiac disease, with familial HOCM accounting for 50% of cases [9]. This disorder is associated with more than one thousand mutations in ten or more genes associated with the cardiac sarcomere [6]. Among the most frequent of these mutations are changes in myosin binding protein C (*MYBPC3*) and myosin-7 (MYH7) [10]. Around 90% of these mutations are missense, involving one normal amino acid being exchanged for another, but some less common cases involve frameshift mutations, which lead to a profoundly different amino acid product [11].

Studies have been conducted on groups of unrelated patients with missense mutations, which yield a truncated protein to find any correlation with prognosis [11]. However, the findings have led to the consensus that a specific mutation cannot be used to successfully determine the disease course nor health outcomes [11]. On the contrary, data from extensive gene sequencing have shown preliminary evidence of compound pathogenic mutations, such as frameshift, being associated with more severe, clinical outcomes including advanced heart failure and sudden death [11]. It is likely that this observation correlates with other genetic diseases in which the severity of disease is often more associated with the degree of deviation from the normal gene product. This is to say that clinical outcomes are more severe in frameshift and nonsense mutations than in missense alterations.

However, genotypic evidence in the absence of phenotypic manifestations of HOCM presents a diagnostic dilemma. It is important to consider the risk of development of HOCM with time, particularly as the age of onset is most common during adolescence. That said, in the presence of a known HOCM genotype, surveillance efforts may be more comprehensive in those at risk. Further, those carrying HOCM genotypes are at risk of producing progeny that are more likely to suffer from HOCM. Though penetrance is not complete, the risk of heritability of this autosomal dominant condition warrants exhaustive genetic counselling with known carriers, affected individuals, and first-degree relatives of those affected.

### 2.2. Diagnosis of HOCM

Hypertrophic obstructive cardiomyopathy is the thickening of the cardiac ventricles or septum which leads to increased effort in pumping blood due to narrowing of the ventricle [12]. The diagnosis of HOCM may be suspected clinically by auscultation of the characteristic harsh systolic murmur, best heard at the lower left sternal border that increases with the Valsalva maneuver without radiation to the carotids [3]. Further, electrocardiogram (EKG) may reveal HOCM by demonstrating left axis deviation or left ventricular hypertrophy. Ultimately, suspicion of this condition is most commonly evaluated with echocardiography ultrasound by which the thickness of the ventricle can be measured. However, due to the limitations of echocardiography by the acoustic window, this technique may be unable to provide a complete depiction of the endocardial border and runs the risk of false-negative findings. In the case of inconclusive echocardiography findings, a cardiac MRI should be performed, as it provides a multiplanar view, coverage of the myocardium without obliquity, and soft-tissue contrast between the myocardial border and blood pool which yields a more accurate analysis of LV hypertrophy [13]. In cases where the patient has a contraindication, a multidetector CT scan should be performed instead of a cardiac MRI [13]. There is a broad range of hypertrophy in patients with HOCM, ranging from 15 mm left ventricle wall thickness to up to 60 mm in some patients [9]. A finding of maximal wall thickness greater than 30 mm is associated with increased risk of sudden cardiac death [9]. However, wall thickness is not the greatest predictor for sudden cardiac death in those affected by HOCM. Rather, this catastrophic event more frequently occurs in young asymptomatic patients predominantly during or after strenuous exercise, hypothesized to occur in association with malignant arrhythmias [9]. People with first-degree relatives diagnosed with HOCM also are at a higher risk and are advised to have a genetic diagnostic test done for the presence of alleles associated with HOCM. If genetic testing services are not feasible, teens between the ages of 12 and 18 should have a yearly echocardiogram and all adults over 18 years of age may have a study completed every five years [9].

### 2.3. Treatment of HOCM

Currently, the standard of treatment for HOCM, aside from managing cardiac risks, is septal myectomy [14]. In this operation, the myocardium of the affected ventricle is physically excised to prevent obstruction of the ventricular outflow tract, hereby decreasing afterload and decreasing cardiac work. In this manner, the operation preserves cardiac function in what would otherwise be a persistently stressed heart. An alternative approach is alcohol septal ablation. This less invasive approach has demonstrated positive outcomes in adults, including low rates of sudden death, sustained decrease in septal thickness and ventricular outflow gradient, and improved functional status [14]. Further, both short-term and long-term outcomes are promising, as rates of reoperation, operative morbidity, and mortality are low while survival is nearly equivalent to that of the general population [14].

While surgical and percutaneous treatments for HOCM have garnered numerous research opportunities, the majority of patients are managed pharmacologically [15]. The use of beta-adrenergic blockade has proven to be most effective in patients with exercise-related dyspnea and angina pectoris associated with left ventricular outflow obstruction by decreasing nonsustained ventricular arrhythmias [15]. However, there remains no evidence of these medications having any impact on long-term patient outcomes [15]. In cases of obstruction at rest, disopyramide has been shown to be effective short-term, but long-term studies reveal that its clinical benefits decrease over time and its use is deterred by its significant anticholinergic side effects [15]. Other drugs, including amiodarone, angiotensin-converting enzyme (ACE) inhibitors, and angiotensin receptor blockers (ARBs), have shown little clinical benefit to patients with HOCM [15]. Thus, surgical and percutaneous treatments remain the most definitive methods for improvement in patient outcomes to date [15].

## 3. Disparities in HOCM

### 3.1. Racial Disparities

Sudden death in competitive athletes is commonly associated with HOCM [4]. This disorder is generally considered rare in the African American population [4]. When investigating the prevalence of HOCM in 286 cases of sudden cardiac death in student athletes between 1985 and 2000, among those in which HCM was identified as the causal etiology, 41% (*n*=42) were white and 55% (*n*=56) were African American [4]. However, in an HCM database of nearly 2000 patients, only 8% were African American [4]. This finding suggests that young African American athletes are less likely to be diagnosed with HOCM and thus are more likely to succumb to this disorder as a cause of sudden cardiac death. A more recent study, however, suggests that the overall incidence of HOCM is indeed higher in African American populations. After multivariate analysis, a 2010 study suggests that African American race is associated with suspected HOCM (OR 4.89, CI 1.24–39.62), when controlled for age, gender, BMI, and hypertension in a random population of 2066 teenagers undergoing screening echocardiography [2]. In contrast, a study evaluating underlying genetic associations with HOCM found that those of African ancestry represented only 5.1% of cases [16]. However, the authors of this 2007 study attribute these findings more so to identification bias rather than an identifiable genetic etiology, suggesting multiple missed opportunities to diagnose this rare condition in a heterogeneous population. In an English study, it is suggested that the groups display largely similar clinical characteristics, though African American patients were more likely to demonstrate EKG abnormality, evidence of left ventricular hypertrophy, and T-wave inversion [17]. Apical hypertrophy and concentric hypertrophy were both more prevalent in black patients [17]. This study demonstrates no differences in survival between races [17]. These data support that a high index of clinical suspicion and routine use of EKG may assist in the timely diagnosis of HOCM in African American populations.

Further, it has been suggested that HOCM is more common in Hispanic children than in Caucasian children [18]. Generally, this relationship is less substantiated than in African American populations. However, there is a growing body of evidence of genetic similarity in the heat-shock proteins that may contribute to the development of HOCM in these two demographics [19]. It is often challenging to study the Latino population, an ethnically and genetically diverse group. However, the dearth of information regarding the incidence of HOCM in this population warrants further investigation for the effective screening and management of affected individuals.

Unfortunately, there is a lack of data regarding the prevalence of HOCM in other minority populations, including Native American, Asian Americans, and Jewish Americans to name a few. It is likely that the absence of data may similarly reflect missed opportunities for diagnosis in these communities. Thus, it is of vital importance that clinicians are cognizant of the possibility of HOCM as a clinical syndrome in all demographics in the United States, as the data support that this condition does not discriminate by race or ethnicity.

### 3.2. Gender Disparities

Few data exist to ascertain the prevalence of HOCM in large populations [20]. However, it is frequently anecdotally reported that HOCM is more common in young men and boys than in their female counterparts. Some data support as high as a 3 : 2 male-to-female predominance [21]. It is hypothesized that this may be attributable to endocrine or genetic factors, particularly due to the effects of estrogen on the myocardium [19]. However, females are more likely to be symptomatic with asymmetric disease [19]. A 2003 investigation describing the population affected with HOCM in New England and the Southwestern United States revealed that the diagnosis of HOCM was more common in boys than in girls [18]. The authors postulate that this observed difference may be attributable to age-related expression of X-linked cardiomyopathies and neuromuscular diseases. Similarly, in a single-center study in South Africa, the HOCM population composition was 62.5% male versus 37.5% female [16]. In a study evaluating HOCM in teenagers, aged 13–19, no statistically significant difference between sexes was observed (*p*=0.3) [2]. A large study of nearly 1000 patients demonstrates that female patients were more likely to be older and more symptomatic than male patients [21]. These women were also more likely to exhibit left ventricular outflow obstruction [21]. Female gender was also associated with risk of progression to Class III or IV heart failure or stroke, particularly in patients older than 50 [21]. This study reveals that the rates of HOCM-associated mortality and sudden death are similar between genders [21]. A meta-analysis of articles through 2015 demonstrates a population that is 37.5% female [22]. This article reveals that women are more likely to be symptomatic and demonstrate higher risk of progression of disease to heart failure or death [22]. Genetic changes in both heat-shock proteins and tumor necrosis factor-*α* predispose women to earlier age of onset and further substantiate the effect of sex hormones on protein expression which may contribute to the development of HOCM [19]. The conflicting data on the prevalence in females mandate an equivalent index of suspicion in female patients as that of males in order to equitably screen for this condition.

### 3.3. Disparities in Age

HOCM is often a diagnosis of the young, though with improvements in management and treatment, affected individuals are surviving to adulthood with greater frequency than in decades past. It is estimated that the average age of affected patients is 42 years, though differences in outcomes and risk of sudden cardiac death exist depending on age and age of diagnosis [21, 23]. Older data support that sudden cardiac death is associated with age younger than 14 years [23]. Patients diagnosed in childhood were usually asymptomatic, but demonstrated a 5.9% annual mortality rate [23]. This group was also more likely to have an unfavorable family history, which was associated with a poor prognosis [23]. Individuals diagnosed between ages 15 and 45 demonstrate a 2.5% annual mortality [23]. Those between ages 45 and 60 at diagnosis exhibit a similar annual mortality of 2.6% [23]. Interestingly, the history at the time of diagnosis rather than the changes in symptoms during interval history was more predictive of poor prognosis than any electrocardiographic or hemodynamic measurement [23]. Undoubtedly, this demonstrates a relationship between early diagnosis and management and improved health outcomes. However, it is critical that HOCM is perceived not as a disease of childhood alone, rather, a spectrum of cardiac disease manifesting in different manners across age groups.

### 3.4. Social Determinants

The diagnosis of HOCM requires diligent workup with careful auscultation, electrocardiogram (EKG), and echocardiography. Access of these tools is more likely in the most frequently diagnosed demographic, white males, particularly when the perceived prevalence for this disorder is higher in this group. Limitations including transportation, geography, insurance status, income, and employment may hinder the ability of individuals to obtain access to such services. This effect of the social determinants of health is embodied across the spectrum of cardiovascular disease, in both pediatric and adult pathologies [24]. With regard to HOCM, these barriers exist not only in access to screening, but also in confirmatory testing, management, and treatment. This principle stipulates that those of lower socioeconomic status or those without a primary care pediatrician or access to a pediatric cardiologist are less likely to be effectively screened, thereby falsely lowering the prevalence of HOCM in these populations. The social determinants of health may also play a role in the lack of robust data in certain demographics, including Native Americans and Hispanic Americans. It is only by increasing access to quality healthcare and striving for culturally competent care for all populations that we may work towards health equity in HOCM and all cardiac disease.

## 4. Suggested Approach to Case Finding

Ultimately, it is only by increasing access to these services and increasing clinical suspicion in the aforementioned demographics that the medical community may work towards improving the diagnosis and treatment of HOCM. One such access improvement project has been underway at Nicklaus Children's Hospital in Miami, FL, USA, since 2012 [25]. This initiative provides free EKG testing to student athletes at no cost to improve early detection of HOCM, subsequently connecting affected children with providers and resources to optimize their health. To date, this program has provided screening for more than 23,000 students. This model offers an ideal example by which medical institutions may partner with schools and community resources to prevent tragedy and to improve the health of communities.

Screening EKGs and cardiac history questionnaires are offered through this initiative at Nicklaus Children's Hospital, outpatient centers ranging from south Miami-Dade County to Palm Beach County, health fairs, school events, and athletic events. Generally, children are healthy at the time of presentation. Per the Seattle Criteria for electrocardiographic interpretation, abnormal findings suggestive of HOCM including left ventricular hypertrophy, large Q waves in leads V6 or aVF, or T-wave inversion in the left lateral leads are referred for further workup [26]. This subsequent appointment includes a physical exam and echocardiography. If the echo is negative and the patient is over the age of 18, there is likely no need for continued surveillance. If it is negative and the student athlete has a positive family history or presents in early adolescence, they are followed annually until HOCM has definitely been excluded as a diagnostic possibility. If the echocardiogram is not definitive, further imaging with cardiac MRI is conducted. If echocardiography reveals criteria consistent with HOCM, the patient is followed and managed by the pediatric cardiology team at Nicklaus Children's Hospital.

Though no consensus has been embraced regarding preparticipation screening for student athletes, the EKG standards applied in this program demonstrate the lowest false-positive rate for identifying arrhythmias [27]. Though further data acquisition is warranted, targeted screening at highest risk groups may be the most effective in terms of both cost and health outcomes for preventing sudden cardiac death due to HOCM [27].

## 5. Conclusions and Future Work

It is imperative that clinicians are mindful of the number of cases that are undiagnosed in the African American, Hispanic, and female communities. In this manner, it is critical that screening is directed at these populations for effective identification and management of HOCM, particularly in the medically underserved. As the understanding of the genetic factors that contribute to the pathophysiology of this disorder becomes more robust and treatment outcomes improve, the morbidity and mortality of HOCM will undoubtedly plummet. It is imperative that all of those affected are properly identified and managed by the appropriate health professionals to enable them not only to survive, but also to thrive despite their diagnosis.

Hypertrophic obstructive cardiomyopathy is a condition that is diverse in both its clinical presentation and in the populations that it affects. For the preservation of families and communities, it is critical that all providers offer attentive auscultation and thorough evaluation for all, particularly those seeking clearance for sports participation. It is only by maintaining a high index of suspicion across ethnicity, gender, age, and class that we may truly achieve equity in the diagnosis and treatment of HOCM.
